# GATA6-AS1 via Sponging miR-543 to Regulate PTEN/AKT Signaling Axis Suppresses Cell Proliferation and Migration in Gastric Cancer

**DOI:** 10.1155/2023/9340499

**Published:** 2023-05-26

**Authors:** Yi Jin, Daqing Jiang

**Affiliations:** Department of Breast Surgery, Cancer Hospital of China Medical University, Liaoning Cancer Hospital & Institute, Shenyang, 110001 Liaoning, China

## Abstract

Gastric cancer (GC) is one of the most common and lethal cancers worldwide. In view of the prominent roles of long noncoding RNAs (lncRNAs) in cancers, we investigated the specific role and underlying mechanism of GATA binding protein 6 antisense RNA 1 (GATA6-AS1) in GC. Quantitative real-time polymerase chain reaction (qRT-PCR) detected GATA6-AS1 expression in GC cell lines. Functional assays were conducted to explore the role of GATA6-AS1 in GC. Furthermore, mechanism investigations were implemented to uncover the interaction among GATA6-AS1, microRNA-543 (miR-543), and phosphatase and tensin homolog (PTEN). In the present study, it was found that GATA6-AS1 expression is significantly downregulated in GC cell lines. Functionally, GATA6-AS1 markedly suppresses GC cell growth and migration *in vitro* and *in vivo* tumorigenesis. Besides tumor suppressor, GATA6-AS1 serves as a miR-543 sponge. Specifically speaking, GATA6-AS1 acts as a competing endogenous RNA (ceRNA) of miR-543 to upregulate the expression of PTEN, thus inactivating AKT signaling pathway to inhibit GC progression. In conclusion, this study has manifested that GATA6-AS1 inhibits GC cell proliferation and migration as a sponge of miR-543 by regulating PTEN/AKT signaling axis, offering new perspective into developing novel GC therapies.

## 1. Introduction

Gastric cancer (GC) is the fifth most prevalent malignancy globally with high morbidity and mortality [1]. The risk factors like atrophic gastritis and intestinal metaplasia contribute to the occurrence of GC. In the past few years, the strategies of reducing the incidence of GC have been developed [2]. For instance, Helicobacter pylori (H. pylori) eradication therapy is effective for GC prevention [3]. In addition, apatinib is approved in the treatment of advanced GC [4]. Furthermore, there are an increasing number of scientific researches, reports, and findings about the molecular mechanism of lncRNAs, contributing to the development of targeted therapies for GC.

Long noncoding RNAs (lncRNAs) refer to transcripts with over 200 nucleotides and without the capacity to code proteins [5]. Multiple lncRNAs have been reported to be involved with the pathogenesis and progression of various cancers [6]. GC is no exception. Some lncRNAs exert cancer-promoting functions in GC. For instance, HOTAIR overexpression was manifested to enhance GC cell proliferation and metastasis and shortening overall survival of GC patients [7]. ATB expedites tumor growth in GC [8]. At the same time, some lncRNAs serve as tumor suppressors in GC. For instance, MEG3 was found to target p53 signaling pathway to attenuate the proliferation and metastasis of GC cells [9].

lncRNA GATA binding protein 6 antisense RNA 1 (GATA6-AS1) acts as a tumor suppressor in several cancers [10]. For example, GATA6-AS1 overexpression was confirmed to indicate poor prognosis of lung squamous cell carcinoma [11, 12]. More importantly, Li et al. have elucidated that overexpressed GATA6-AS1 could inhibit LNM and EMT via FZD4 in GC cells by targeting the Wnt/*β*-catenin pathway [13]. It was worth noting that in their report, GATA6-AS1 was located in the nucleus and participated in the regulation of GC at the transcriptional level. However, it was unclear whether GATA6-AS1 regulates the progression of GC via acting as a competing endogenous RNA (ceRNA) at the posttranscription level.

In recent years, ceRNA mechanism has been one of the most popular regulation mechanisms concerning lncRNA, and ceRNA is a new type of gene expression regulation mode [14]. Previous reports have indicated that lncRNA could sponge microRNA (miRNA) to regulate the expression of messenger RNA (mRNA) as a ceRNA, so as to take part in the progression of cancers [15]. For example, XLOC_006390 could promote cervical tumorigenesis by serving as a ceRNA to target miR-331-3p and miR-338-3p [16]. FAL1 was proved to accelerate the proliferative and migratory capacities of hepatocellular carcinoma cells *in vitro* through ceRNA mode with miR-1236 [17].

In this study, we intended to explore whether GATA6-AS1 affects GC progression in terms of ceRNA regulatory mode.

## 2. Materials and Methods

### 2.1. Cell Lines and Reagent

Human gastric mucosa cell line (GES-1) used in this study was procured from BeNa Culture Collection (Beijing, China). Human GC cell lines (HGC-27, AGS) were bought from Cell Bank of the Chinese Academy of Sciences (Shanghai, China); both MKN-7 and MKN-45 cell lines were obtained from Procell Life Science & Technology Co., Ltd. (Wuhan, China). RPMI-1640 commercially acquired from Thermo Fisher Scientific (Waltham, MA) was used to culture GES-1, HGC-27, MKN-7, and MKN-45 cells under 37°C and 5% CO_2_. AGS cells were cultivated in F12K medium (Gibco). In addition, 10% fetal bovine serum (FBS; Gibco) and 1% penicillin-streptomycin (Gibco) both served as medium supplements. SF1670 (1 *μ*M), an inhibitor of PTEN (phosphatase and tensin homolog), was purchased from MedChemExpress (South Brunswick, NJ).

### 2.2. Total RNA Isolation and Quantitative Real-Time Polymerase Chain Reaction (qRT-PCR)

TRIzol reagent purchased from Invitrogen (Carlsbad, CA) was used to isolate total RNAs from the cultured cells. Reverse transcription of RNA into cDNA was conducted via the implementation of PrimeScript™ RT reagent kit (Takara, Shiga, Japan) as per the instructions of manufacturer. Power SYBR® Green Master Mix bought from Applied Biosystems (Carlsbad, CA) was then employed for PCR on the StepOne™ Real-Time PCR System from Applied Biosystems. Relative gene expression was calculated by 2^-*ΔΔ*Ct^ method. GAPDH or U6 was used as the internal reference. Each experiment was undertaken in triplicate, with three technical replicates for each biorepeat.

### 2.3. Plasmid Transfection

The pcDNA3.1/GATA6-AS1 and negative control pcDNA3.1 were available from GenePharma (Shanghai, China) for 48 h transfection into GC cell samples using Lipofectamine 3000 (Invitrogen). For the sake of silencing GATA6-AS1 and PTEN, the short hairpin RNAs (shRNAs) and negative controls of shRNAs (sh-NCs) were also specifically designed by and purchased from GenePharma. In addition, microRNA-543 (miR-543) mimics were transfected into GC cells to overexpress miR-543, while miR-543 inhibitors for silencing miR-543. In this study, miR-543 mimics, miR-543 inhibitors, and corresponding negative controls (NC mimics/NC inhibitors) were purchased from RiboBio (Guangzhou, China) for plasmid transfection.

### 2.4. Cell Counting Kit-8 (CCK-8)

Transfected GC cells were harvested and seeded into 96-well plates. A total of 10 *μ*L CCK-8 (Dojindo Laboratories, Kumamoto, Japan) was then added into each well of the plates for 1 h under culture condition. The absorbance at 450 nm was monitored with a microplate reader. Independent experiment was done in triplicate, with three technical replicates for each biorepeat.

### 2.5. Colony Formation

MKN-45, AGS, and HGC-27 cells were collected at log phase of growth post transfection and subsequently seeded into 6-well plates at a density of 500 cells each well. After 14-day incubation, cells were fixed with 4% paraformaldehyde and then dyed by 0.1% crystal violet. Finally, the colonies in three separately conducted assays were manually counted. Independent experiments were performed in triplicate. Three technical replicates were performed for each biorepeat.

### 2.6. Wound Healing

The processed cell samples were cultured in 96-well plates with culture medium with no serum. After reaching the required confluence, the samples were scratched by pipette tips and photographed immediately (0 h). With the rinse in phosphate-buffered saline (PBS), the wounds were observed 24 h later and photographed by microscopy (Olympus, Tokyo, Japan). Each experiment was carried out in triplicate, with three technical replicates for each biorepeat.

### 2.7. Transwell Migration Assay

Totally, 1 × 10^5^ transfected cell samples were inoculated into the upper part of transwell chamber (Corning, NY). Serum-free medium was added to cultivate the cells in the upper chamber. The complete medium was added to the lower chamber. Prior to 24-hour incubation at 37°C, the migrated cells to the lower chamber were fixed and dyed by 0.5% crystal violet. Inverted microscope (Olympus) was employed to count the stained cells in 5 random fields. Separate experiment was undertaken in triplicate, with three technical replicates for each biorepeat.

### 2.8. RNA Pull-Down Assay

For RNA pull-down assay, specific miR-543 probe was synthesized and biotinylated, forming wild-type and mutant Bio-miR-543 probes (Bio-miR-543-WT/Mut). Bio-miR-543-WT/Mut probes were then incubated with the protein extracts from GC cells along with magnetic beads. Subsequent to RNA isolation, the purified RNA from the collected pull-down complex was assayed using qRT-PCR to detect relative RNA enrichment. Each experiment was undertaken in triplicate, with three technical replicates for each biorepeat.

### 2.9. RNA-Binding Protein Immunoprecipitation (RIP)

RNA interaction was examined by RIP assay in GC cells using Magna RIP™ RNA-Binding Protein Immunoprecipitation Kit (Millipore, Billerica, MA) in line with the supplier's suggestions. Subsequent to the lysis in RIP lysis buffer, cell lysates were treated with human Argonaute2 (Ago2) antibody (anti-Ago2, 1/1,000–1/2,000, ab186733; Abcam, Cambridge, MA) or control immunoglobulin G antibody (anti-IgG, 1/1,000–1/10,000, ab133470; Abcam). Followed by the addition of magnetic beads, qRT-PCR was performed after RNA purification and RNA extraction. Each experiment was undertaken three times, with three technical replicates for each biorepeat.

### 2.10. Western Blot

Cell protein samples extracted from cultured cells were prepared for the electrophoresis separation using 12% sodium dodecyl sulphate-polyacrylamide gel electrophoresis (SDS-PAGE). Subsequently, the samples were shifted onto polyvinylidene fluoride (PVDF) membranes. The diluted primary antibodies against PTEN (1 : 10,000, ab32199; Abcam) and loading control GAPDH (1 : 1,000, ab8245; Abcam) were incubated with the blocked membranes overnight at 4°C. After washing in tris-buffered saline-tween (TBST), the membranes were subjected to incubation with the secondary antibodies tagged with horseradish peroxidase (1 : 5,000, ab7090; Abcam). ECL luminous liquid bought from Pierce (Rockford, IL) was applied for the detection of proteins on western blots. Each experiment was undertaken in triplicate, with three technical replicates for each biorepeat.

### 2.11. Subcellular Fractionation Assay

The subcellular fractionation assay was performed with the application of PARIS™ kit acquired from Ambion (Austin, TX) following the guidebook. AGS and MKN-45 cells (1 × 10^6^ cells) were harvested and placed on ice after PBS washing. The precooled cell fractionation buffer was added to cell samples for 10 min incubation. Following centrifugation for 5 min, cytoplasmic and nuclear fractions were separated. GATA6-AS1 expression was detected by qRT-PCR analysis. Each experiment was undertaken in triplicate, with three technical replicates for each biorepeat.

### 2.12. Fluorescence *In Situ* Hybridization (FISH)

The specific probe for GATA6-AS1 was designed and synthesized by RiboBio for FISH assay. GC cells were fixed for 20 min, followed by the treatment with protease K for 10 min. After being washed in PBS, the probe and hybridization buffer were added to cell samples overnight at 42°C. The fluorescence detection was undertaken with Olympus fluorescence microscope. Each experiment was undertaken in triplicate, with three technical replicates for each biorepeat.

### 2.13. Luciferase Reporter Assay

The wild-type and mutant GATA6-AS1 or PTEN-3′ untranslated region (3′-UTR) fragments containing the potential sites for the binding of miR-543 were acquired and inserted into luciferase vector pmirGLO, establishing luciferase reporter vectors including GATA6-AS1-WT/Mut and PTEN-3′-UTR-WT/Mut. The acquired constructs were then cotransfected into HEK-293T cells (ATCC, Manassas, VA) with indicated plasmids for 48 h. At length, Luciferase Reporter Assay System (Promega, Madison, WI) was employed for examining the luciferase intensity as instructed. Each experiment was undertaken in triplicate, with three technical replicates for each biorepeat.

### 2.14. *In Vivo* Experiments

A total of 15 BALB/c nude mice (6 weeks old, male) purchased from Model Animal Research Center of Nanjing University were prepared for the animal experiments. Animal study was approved by the Institutional Animal Care and Use Committee of Cancer Hospital of China Medical University. GATA6-AS1-overexpressed or GATA6-AS1-overexpressed+PTEN-silenced MKN-45 cells were used to treat nude mice with subcutaneous injection. The mice treated with pcDNA3.1-transfected cells were used as negative controls. The volume of tumors was examined every 4 days. Four weeks post subcutaneous injection, the nude mice were sacrificed, and the xenografted tumors were resected for the measurement.

### 2.15. Statistical Analysis

Each assay in the study was biorepeated in triplicate. The experimental data were expressed as the mean ± standard deviation (S.D.) and processed by Prism 6 (GraphPad, San Diego, CA). The *P* value below 0.05 was defined as significant difference for the statistical analyses. Student's *t*-test or analysis of variance (one-way/two-way ANOVA) was used for comparing differences between groups.

## 3. Results

### 3.1. GATA6-AS1 Is Lowly Expressed in GC Tissues and Cell Lines

**Figure 1:**
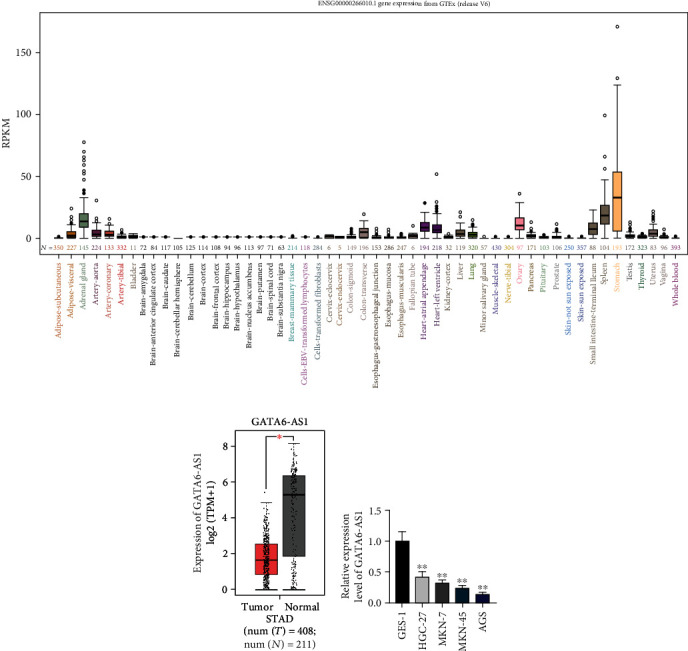
GATA6-AS1 is lowly expressed in the GC tissues and cell lines. (a) UCSC database displayed the level of GATA6-AS1 in human normal tissues. GATA6-AS1 expression level was significantly elevated in normal stomach tissues. (b) GEPIA analysis displayed the GATA6-AS1 expression in tumor (T; *n* = 408) and normal (N; *n* = 211) tissues. (c) GATA6-AS1 expression in GES-1 and HGC-27, MKN-7, MKN-45, and AGS cell lines was detected by qRT-PCR. One-way ANOVA and Dunnett's test. ^∗∗^*P* < 0.01 and ^∗^*P* < 0.05.

To detect the biological function of GATA6-AS1 in GC, we analyzed GATA6-AS1 expression in normal tissues and tumor tissues through UCSC and TCGA databases. UCSC database (http://genome.ucsc.edu/) showed that GATA6-AS1 expression was the highest in the normal stomach tissues compared with the other normal tissues (Figure 1(a)). In addition, TCGA database (http://gepia2.cancer-pku.cn/#index) displayed that GATA6-AS1 was significantly underexpressed in stomach adenocarcinoma (STAD) tissues compared with that in normal tissues (Figure 1(b)). Besides, qRT-PCR was applied to measure the GATA6-AS1 expression in GC cell lines (HGC-27, MKN-7, MKN-45, and AGS) relative to normal cell line. The results elucidated that the GATA6-AS1 was expressed at low level in GC cell lines versus that in GES-1 (Figure 1(c)). We also found that GATA6-AS1 expression was relatively higher in HGC-27 cell line but relatively lower in AGS and MKN-45 cell lines, so we chose these three cells for subsequent experiments. Taken together, GATA6-AS1 is underexpressed in GC tissues and cell lines.

### 3.2. GATA6-AS1 Represses Cell Proliferation and Migration in GC and Suppresses *In Vivo* Tumorigenesis

**Figure 2:**
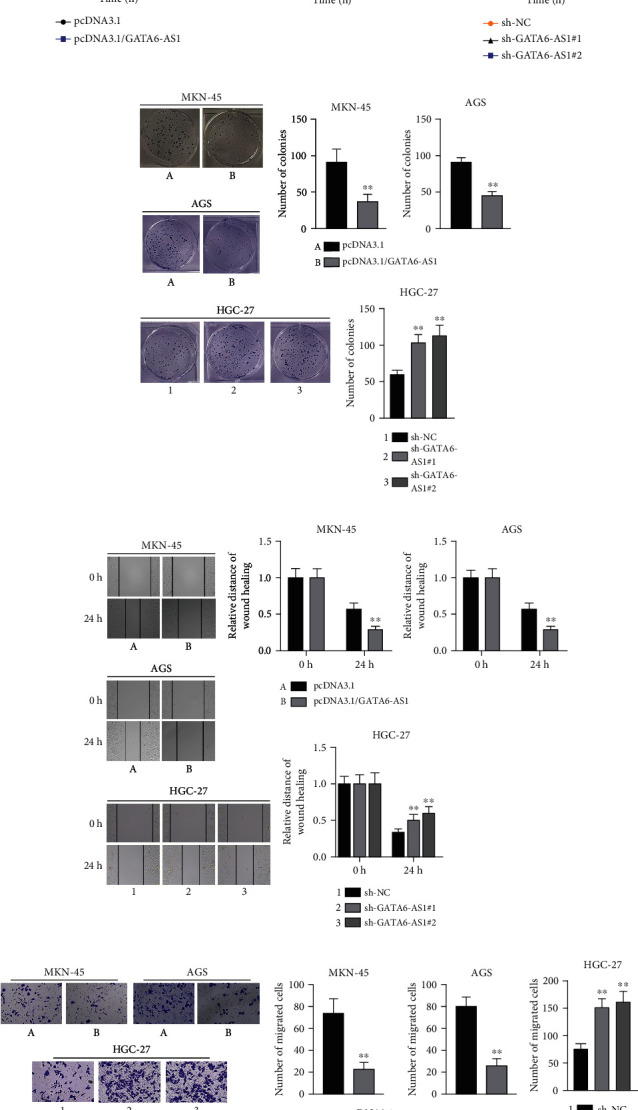
GATA6-AS1 represses cell proliferation and migration in GC. (a) QRT-PCR was used to analyze the overexpression efficiency of pcDNA3.1/GATA6-AS1 in transfected MKN-45 and AGS cells and interference efficiency of sh-GATA6-AS1#1/#2/#3 in transfected HGC-27 cells. (b) CCK-8 assays were applied to access the cell viability when GATA6-AS1 was overexpressed or inhibited in GC cells. (c) Colony formation assays were performed to assess cell proliferation when GATA6-AS1 was upregulated or silenced in GC cells. (d) The migration ability of GC cells was assessed by wound healing assays in the indicated cells. (e) Transwell assays were further conducted to evaluate cell migration after the overexpression or deficiency of GATA6-AS1 in GC cells. (f–h) Representative image, tumor growth curve, and tumor weight at the end points of xenografted tumors formed by subcutaneous injection of MKN-45 cells stably transfected with pcDNA3.1 or pcDNA3.1/GATA6-AS1 into nude mice. The number of nude mice used in each group is 3. Student's *t*-test for overexpression studies and one-way ANOVA and Dunnett's test for knockdown studies. ^∗∗^*P* < 0.01.

Next, we knocked down GATA6-AS1 in HGC-27 cells and overexpressed GATA6-AS1 in MKN-45 and AGS cells. After the transfection of pcDNA3.1/GATA6-AS1 into or the transfection of sh-GATA6-AS1#1/#2/#3 into GC cells, qRT-PCR analysis was applied to test the overexpression efficiency of pcDNA3.1/GATA6-AS1 or silencing efficiency of sh-GATA6-AS1#1/2/3. The results demonstrated that GATA6-AS1 expression was efficiently increased by pcDNA3.1/GATA6-AS1 in comparison with control (Figure 2(a), left panels). The level of GATA6-AS1 was obviously diminished by the transfection of sh-GATA6-AS1#1/#2/#3 into HGC-27 cells (Figure 2(a), right panel). Particularly, sh-GATA6-AS1#1 and sh-GATA6-AS1#2 presented a relatively higher knockdown efficiency (Figure 2(a), right panel). Subsequently, we chose pcDNA3.1/GATA6-AS1 and sh-GATA6-AS1#1/#2 for the following investigations.

For further study on the effects of GATA6-AS1 on the malignant progression of GC, we designed functional assays to assess the function of GATA6-AS1 in GC cells. With CCK-8 and colony formation assays examining the proliferation of GC cells, we discovered that the absorbance at 450 nm in GC cells was markedly declined after the transfection with pcDNA3.1/GATA6-AS1 in comparison with negative control (Figure 2(b), left panels), while that was significantly increased in the HGC-27 cells transfected with sh-GATA6-AS1#1 and sh-GATA6-AS1#2 compared with the sh-NC group (Figure 2(b), right panel), indicating that overexpressed GATA6-AS1 restrains cell viability while GATA6-AS1 depletion promotes that in GC. Besides, colony formation assay displayed that overexpression of GATA6-AS1 reduced the number of colonies but knockdown of GATA6-AS1 increased that, which indicated that upregulation of GATA6-AS1 hampers GC cell proliferation (Figure 2(c), upper panels) but downregulation of GATA6-AS1 enhances that (Figure 2(c), lower panels). In addition, wound healing and transwell assays were implemented to assess the capacity of GC cells to migrate. The results of wound healing assays presented that the relative distance of wound healing at 24 h in the pcDNA3.1/GATA6-AS1 group was evidently narrower versus the control group (Figure 2(d), upper panels), while that in the sh-GATA6-AS1#1/#2 groups at 24 h was wider versus the control group (Figure 2(d), lower panels), indicating that GATA6-AS1 overexpression inhibits cell migration while GATA6-AS1 knockdown propels that. The results of transwell assay also verified this finding as overexpression of GATA6-AS1 effectively decreased the number of migrated cells (Figure 2(e), upper left panels and middle panels) while knockdown of GATA6-AS1 increased the number of migrated cells (Figure 2(e), lower left panels and right panel). Consistently, the injection of pcDNA3.1/GATA6-AS1 transfected cells resulted in a marked decrease in the growth of xenografted tumors (Figures 2(f)–2(h)).

Overall, GATA6-AS1 represses the abilities of GC cells to proliferate and migrate *in vitro* and suppresses *in vivo* tumorigenesis.

### 3.3. GATA6-AS1 Binds to miR-543 in GC Cells

**Figure 3:**
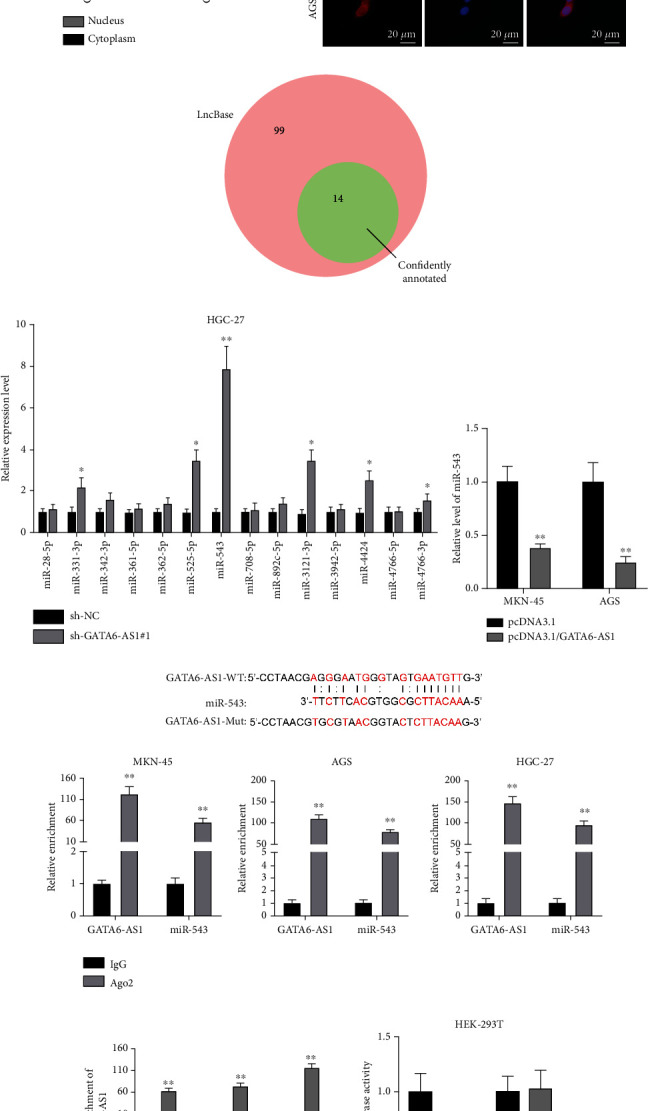
GATA6-AS1 binds to miR-543 in GC cells. (a, b) Cytoplasmic/nuclear fractionation and FISH assays were performed to detect the subcellular distribution of GATA6-AS1 in MKN-45 and AGS cells. U6 and GAPDH were used as control. Scale bar = 20 *μ*m. (c) LncBase database was used to screen out 14 putative target miRNAs which possibly bind to GATA6-AS1 and are confidently annotated. (d) The levels of 14 candidate miRNAs in GATA6-AS1-knockdown HGC-27 cells were detected by qRT-PCR. (e) miR-543 level in GATA6-AS1-overexpression MKN-45 and AGS cells was detected via qRT-PCR. (f) The starBase predicted the potential binding site between GATA6-AS1 and miR-543. The sequence of GATA6-AS1 was mutated based on the miR-543 binding site to obtain the GATA6-AS1-Mut plasmid. (g) The enrichment of GATA6-AS1 and miR-543 in anti-Ago2 bound complex was detected by RIP assays. (h) RNA pull-down assays followed by qRT-PCR analysis were carried out to detect the expression of GATA6-AS1 in the complexes pulled down by wild-type biotinylated miR-543 (Bio-miR-543-WT) or mutant biotinylated miR-543 (Bio-miR-543-Mut). (i) Luciferase reporter assays were conducted to further verify the interaction of GATA6-AS1 and miR-543. Luciferase activity of reporter vector containing wild-type GATA6-AS1 (GATA6-AS1-WT) or mutant type (GATA6-AS1-Mut) was detected post cotransfection with miR-543 mimics or NC mimics into HEK-293T cells. Student's *t*-test was used in (d), (e), (g), and (i) and one-way ANOVA and Dunnett's test for (h). ^∗^*P* < 0.05 and ^∗∗^*P* < 0.01.

Next, we investigated the regulatory mechanism of GATA6-AS1 in GC. Previous studies have reported that lncRNAs participate in the biological processes via regulating downstream target genes [18] and some sponge miRNAs to regulate mRNA expression by acting as a ceRNA [14]. ceRNA network is a posttranscriptional regulatory mechanism concerning cytoplasmic lncRNA [19]. Thus, we firstly detected the subcellular localization of GATA6-AS1 in GC cells. Through subcellular fractionation assays, we discovered that GATA6-AS1 is localized in both cytoplasm and nucleus of GC cells (Figure 3(a)). FISH assay also detected GATA6-AS1 fluorescence (red) in both nucleus and cytoplasm (Figure 3(b)). Given that, we hypothesized that GATA6-AS1 might regulate the development of GC at the transcriptional and posttranscriptional levels. Considering that the transcriptional regulation of GATA6-AS1 in GC has been uncovered [13], we decided to explore its posttranscriptional regulation mechanism in terms of ceRNA network. As Figure 3(c) presented, ninety-nine downstream miRNAs of GATA6-AS1 were screened out on the LncBase database via DIANA (http://carolina.imis.athena-innovation.gr/diana_tools/web/index.php?r=lncbasev2%2Findex-predicted). Notably, fourteen miRNAs have been confidently annotated and have been studied by other researchers before. To further determine the most suitable target miRNA of GATA6-AS1 in GC cells, qRT-PCR was applied to test the levels of candidate miRNAs in the HGC-27 cells transfected with sh-GATA6-AS1#1. The results revealed that the level of miR-543 was the most upregulated among candidate miRNAs (Figure 3(d)). Thus, we selected miR-543 as a potential target of GATA6-AS1 for the follow-up exploration. To further determine the association between GATA6-AS1 and miR-543, we detected miR-543 level in GATA6-AS1-overexpression GC cells via qRT-PCR analysis. It showcased that the level of miR-543 was downregulated in GATA6-AS1-overexpression GC cells (Figure 3(e)), indicating that GATA6-AS1 negatively regulates miR-543 expression. Further, we performed mechanism investigation to verify whether GATA6-AS1 binds to miR-543 in GC cells. As Figure 3(f) presented, the starBase database (https://starbase.gene.com/) predicted the potential binding sites between GATA6-AS1 and miR-543. The results of RIP displayed that GATA6-AS1 and miR-543 were both overtly enriched in the Anti-Ago2 groups versus that in Anti-IgG controls in GC cells (Figure 3(g)), indicating the existence of GATA6-AS1 and miR-543 in RNA-induced silencing complex (RISC) as Ago2 is the main component of RISC. Additionally, RNA pull-down assay further uncovered that GATA6-AS1 was highly enriched in the Bio-miR-543-WT groups while GATA6-AS1 had no marked abundance in the Bio-miR-543-Mut groups relative to controls (Figure 3(h)), indicating that GATA6-AS1 binds to miR-543 in GC cells. Moreover, the luciferase reporter assays further verified this finding as overexpression of miR-543 led to a remarkable decline of luciferase activity in the GATA6-AS1-WT groups compared to the NC mimics groups, while no marked changes were found in the GATA6-AS1-Mut groups (Figure 3(i)). All in all, GATA6-AS1 binds to miR-543 in GC cells.

### 3.4. GATA6-AS1 Represses Cell Growth and Migration by Regulating miR-543 in GC

**Figure 4:**
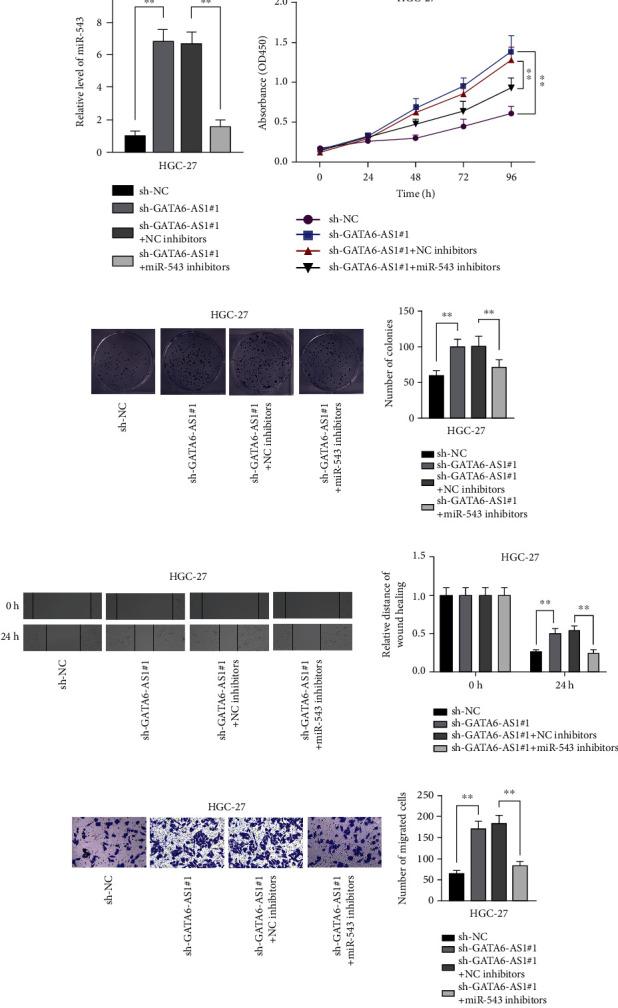
GATA6-AS1 represses cell growth and migration by regulating miR-543 in GC. (a) QRT-PCR was applied to estimate miR-543 level in HGC-27 cells transfected with sh-GATA6-AS1#1 and miR-543 inhibitors. (b) GC cell proliferation was estimated by CCK-8 assays when GATA6-AS1 and miR-543 were silenced. (c) Colony formation assays measured cell proliferation when GATA6-AS1 and miR-543 were knocked down. (d, e) The migratory capacity was assessed by wound healing and transwell assays when GATA6-AS1 and miR-543 were inhibited. One-way ANOVA and Dunnett's test. ^∗∗^*P* < 0.01.

To further study the effect of GATA6-AS1-miR-543 interaction on GC cell behaviors, we performed several relevant functional assays along with rescue experiments. Before that, we applied qRT-PCR to detect the level of miR-543 in HGC-27 cells transfected with sh-NC, sh-GATA6-AS1#1, sh-GATA6-AS1#1+NC inhibitors, or sh-GATA6-AS1#1+miR-543 inhibitors. The results showed that miR-543 level was conspicuously upregulated in GATA6-AS1-knockdown HGC-27 cells and dramatically decreased by the additional transfection of miR-543 inhibitors (Figure 4(a)). CCK-8 assays revealed that the increase in cell viability caused by GATA6-AS1 deficiency could be fully reversed by cotransfection with miR-543 inhibitors (Figure 4(b)), indicating that miR-543 reversed the increase in cell viability induced by GATA6-AS1 knockdown and miR-543 silencing represses cell viability in GC cells. Likewise, the number of colonies was increased by GATA6-AS1 depletion but reduced by miR-543 silencing (Figure 4(c)), which indicated that cell proliferation was enhanced by inhibited GATA6-AS1 but reversed by silenced miR-543. Furthermore, via wound healing assay, we discovered that the relative distance of wound healing was increased in the sh-GATA6-AS1#1 groups compared to control and then altered by the inhibition of miR-543 (Figure 4(d)), indicating that miR-543 inhibition reverses the promoting effect of silenced GATA6-AS1 on the migratory ability of GC cells. Besides, transwell assay also indicated that downregulation of miR-543 could offset the promoting function of inhibited GATA6-AS1 on cell migration (Figure 4(e)). On the whole, GATA6-AS1 represses the capacities of GC cell to proliferate and migrate via modulating miR-543.

### 3.5. PTEN Is the Target Gene of miR-543 in GC Cells

**Figure 5:**
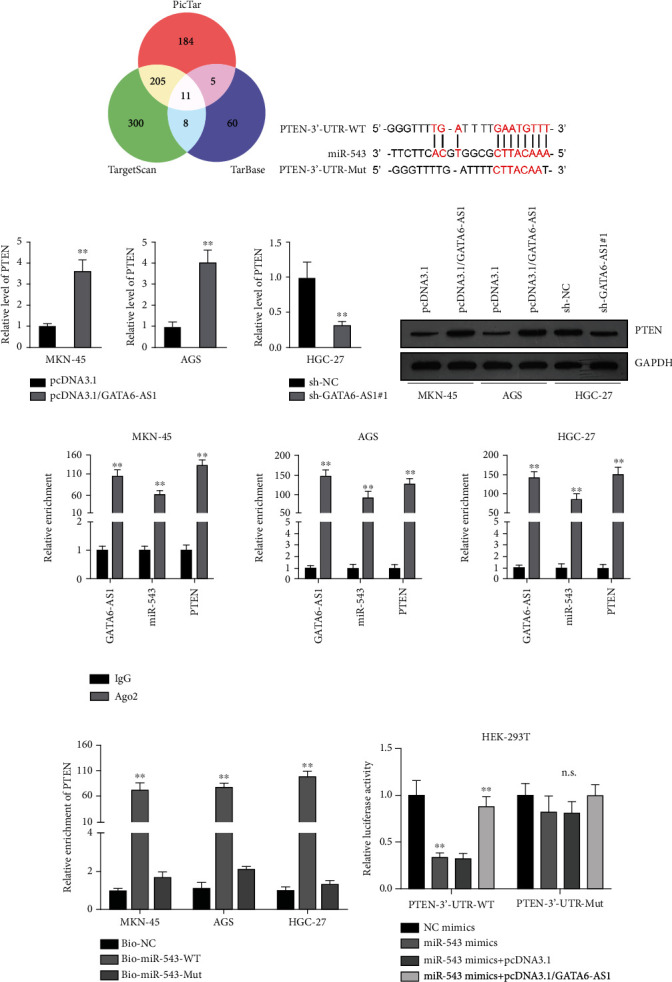
PTEN is the target gene of miR-543 in GC cells. (a) Venn diagram represented the overlap of miR-543 targets based on three algorithms (PicTar, TargetScan, and TarBase). 11 predicted miR-543 targets are TWIST1, DYNC1LI2, PTEN, HERPUD1, FNDC3B, FOXP1, ANKRD13C, BTBD3, CDH11, ZNF281, and COPS2. (b) The starBase was utilized to predict the potential binding site between miR-543 and PTEN-3′-UTR. The sequence of PTEN-3′-UTR-WT containing miR-543-binding site was mutated to obtain PTEN-3′-UTR-Mut plasmid. (c) The level of PTEN in the indicated GC cells after the upregulation or depletion of GATA6-AS1 was assessed via qRT-PCR. (d) Western blot analysis was utilized to measure the protein level of PTEN in the transfected cells after the overexpression and downregulation of GATA6-AS1. (e) RIP assays were conducted to analyze the association of GATA6-AS1, miR-543, and PTEN in GC cells. (f) RNA pull-down assays verified the binding between miR-543 and PTEN. (g) Luciferase activity of PTEN-3′-UTR was detected. Student's *t*-test was used in (c)–(e) and one-way ANOVA followed by Dunnett's test for (f) and (g). ^∗∗^*P* < 0.01. n.s.: no significance.

To further probe into the regulatory mechanism of GATA6-AS1 in GC, we utilized bioinformatics to predict potential targets of miR-543. The putative mRNAs of miR-543 were obtained according to the combined prediction of PicTar, TargetScan, and TarBase databases (Figure 5(a)). Among 11 putative mRNAs, PTEN has been extensively reported to exert tumor-suppressive function in multiple cancers [20–22]. Thereby, PTEN was chosen for the follow-up investigations. Further, starBase was utilized to predict the potential binding sites, which suggested that miR-543 had the potential to bind to PTEN-3′-UTR (Figure 5(b)). In addition, we investigated the regulatory relationship between GATA6-AS1 and PTEN via qRT-PCR analysis of PTEN levels in GC cells after the overexpression or depletion of GATA6-AS1. The results showed that PTEN level was apparently enhanced by GATA6-AS1 overexpression in GC cells relative to controls (Figure 5(c), upper panel and left panel) but markedly declined by GATA6-AS1 deficiency (Figure 5(c), right panel), indicating that GATA6-AS1 positively regulates PTEN expression in GC cells. Western blot analysis also verified this finding as PTEN protein level was notably increased in GATA6-AS1-overexpression MKN-45 and AGS cells but reduced in GATA6-AS1-silence HGC-27 cells (Figure 5(d)). RIP assays were conducted to analyze the interaction among GATA6-AS1, miR-543, and PTEN in GC cells. It displayed that GATA6-AS1, miR-543, and PTEN were all significantly abundant in the Anti-Ago2 groups versus those in Anti-IgG controls (Figure 5(e)), suggesting that GATA6-AS1, miR-543, and PTEN coexist in Ago2-RISC. Then, RNA pull-down assays were conducted to further prove the interaction between miR-543 and PTEN. The results showed that PTEN was obviously enriched in the Bio-miR-543-WT groups while the Bio-miR-543-Mut groups had no significant abundance of PTEN compared with controls (Figure 5(f)), suggesting that miR-543 could bind to PTEN in GC cells. Subsequently, luciferase reporter assay was employed to detect the interaction between GATA6-AS1, miR-543, and PTEN. The results showed the decline on the luciferase activity of PTEN-3′-UTR-WT by miR-543 mimics and then recovered by overexpressed GATA6-AS1 (Figure 5(g)). However, the PTEN-3′-UTR-Mut groups had no remarkable change (Figure 5(g)). Taken together, PTEN serves as a direct target of miR-543 in GC cells.

### 3.6. GATA6-AS1 Suppresses the Progression of GC by Regulating PTEN/AKT Signaling Axis

**Figure 6:**
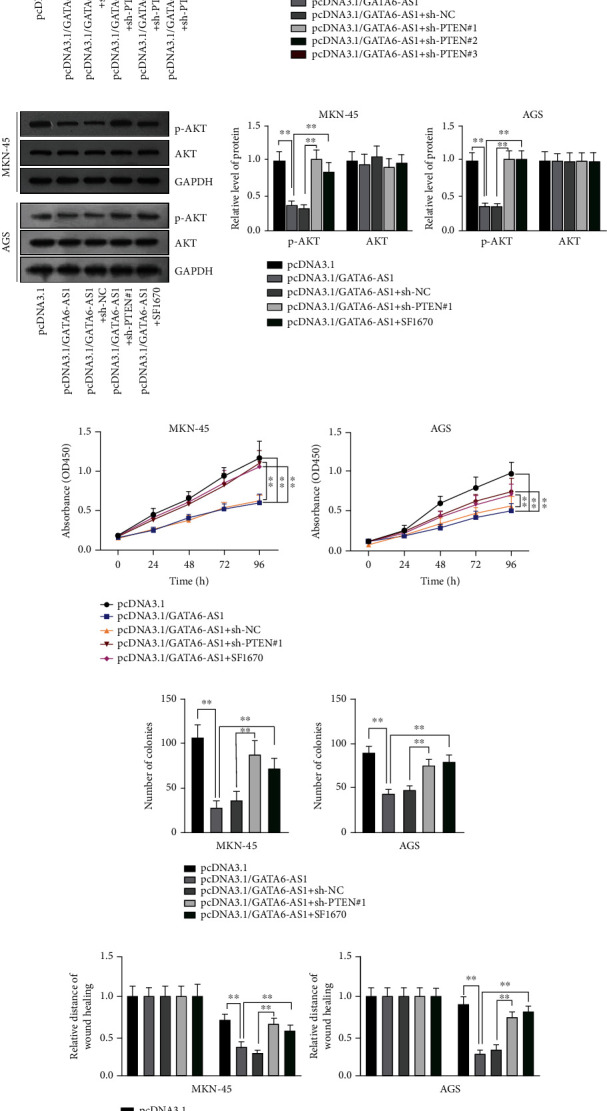
GATA6-AS1 suppresses the progression of GC by regulating PTEN/AKT signaling axis. (a) QRT-PCR was implemented to detect PTEN level in GC cells transfected with pcDNA3.1/GATA6-AS1 and sh-PTEN#1/#2/#3. (b) Western blot was applied to evaluate the protein level of PTEN in GC cells after the transfection with pcDNA3.1/GATA6-AS1 and sh-PTEN#1/#2/#3 (left panels). Quantification of western blot results was shown in the bar graphs (right panels). (c) Western blot was applied to detect the protein level of AKT and phosphorylated AKT (p-AKT) in the transfected cells with overexpressed GATA6-AS1 and inhibited PTEN or added with SF1670 (left panels). Quantification of western blot results was shown in the bar graphs (right panels). (d) Cell viability of GC cells in different groups was assessed by CCK-8. (e) Cell proliferation in different groups was evaluated by colony formation assays. (f) Wound healing assays were conducted to assess the migratory capacity of GC cells in different groups. (g) The evaluation of cell migration in different groups through transwell assays was displayed. One-way ANOVA followed by Dunnett's test. ^∗∗^*P* < 0.01.

Finally, we assessed the effect of PTEN-GATA6-AS1 interaction in GC cells. Based on qRT-PCR results, PTEN expression was increased after the overexpression of GATA6-AS1 and downregulated after the knockdown of PTEN in GC cells (Figure 6(a)). Additionally, western blot also indicated that PTEN protein levels were elevated when GATA6-AS1 was overexpressed and declined after the additional transfection with sh-PTEN#1/#2/#3 (Figure 6(b)). Afterwards, western blot analyses were implemented to determine whether PTEN knockdown activates AKT function. The phosphorylated AKT (p-AKT) level was reduced after the overexpression of GATA6-AS1 but upregulated by the silence of PTEN or addition of SF1670 (Figure 6(c)). SF1670 was a specific PTEN inhibitor, which could also accelerate the phosphorylation of AKT indirectly. In this assay, we also discovered that the repressed phosphorylation of AKT was activated by SF1670 (Figure 6(c)). According to CCK8 assays, it was found that enhanced GATA6-AS1 suppressed cell viability, while silencing PTEN or adding SF1670 could recover cell viability in GC (Figure 6(d)). Then, colony formation assays showed that colonies were reduced by upregulated GATA6-AS1, but this was reversed by silenced PTEN or addition of SF1670, which indicated that cell proliferation could be restrained by GATA6-AS1 overexpression but reversed by the depletion of PTEN or addition of SF1670 (Figure 6(e)). Moreover, wound healing assays assessed cell migration in GC. The outcomes suggested that the relative distance of wound healing was decreased by GATA6-AS1 overexpression but then increased by PTEN depletion or addition of SF1670 (Figure 6(f)). Finally, transwell assays also verified that the decreased number of migrated cells caused by GATA6-AS1 upregulation could be offset by the ablation of PTEN or addition of SF1670 (Figure 6(g)). Taken together, GATA6-AS1 could suppress cell proliferation and migration in GC by regulating PTEN/AKT signaling axis.

### 3.7. GATA6-AS1 Represses In Vivo GC Tumorigenesis via Regulating PTEN

Besides *in vitro* assays, *in vivo* experiments were performed to verify this molecular mechanism. Xenograft mouse model was established by subcutaneous injection of GATA6-AS1-overexpressed or GATA6-AS1-overexpressed+PTEN-inhibited MKN-45 cells into nude mice, with those injected with pcDNA3.1 as controls. It was found that the volume and weight of xenografts were significantly reduced in the pcDNA3.1/GATA6-AS1 group versus the control group but cotransfection of sh-PTEN#1 altered the suppressive effect of GATA6-AS1 overexpression on tumor growth (Figure S1A-C). Further, it was found that GATA6-AS1 positively regulates PTEN expression *in vivo* (Figure S1D-E). To conclude, GATA6-AS1 represses *in vivo* GC tumor growth via regulating PTEN.

## 4. Discussion

lncRNAs, associated with different types of cancers, regulate gene expression via modulating transcription and chromatin modification [23]. Besides the promoting effects, lncRNAs have the suppressive effects on cancer progression [24]. Previous studies have reported that upregulated GATA6-AS1 predicts poor prognosis of lung squamous cell carcinoma [11, 12]. GATA6-AS1 impedes the progression of non-small-cell lung cancer [1]. Moreover, GATA6-AS1 has been found to be lowly expressed in GC, and upregulated GATA6-AS1 has been elucidated to suppress the progression of GC by downregulating FZD4 expression to inactivate the Wnt/*β*-catenin signaling pathway [13]. With the induction of GATA6-AS1 depletion, miR-582 facilitated liver and lung metastasis of GC via downregulating FOXO3 expression and promoting the activity of PI3K/AKT/Snail pathway [25]. Apart from the regulation of cancer development, GATA6-AS1 also exerts function in normal tissues. For instance, GATA6-AS1 facilitates human endoderm differentiation through upregulating GATA6 expression by promoting SMAD2/3-mediated GATA6 transcriptional activation [26].

In the present study, we discovered that GATA6-AS1 expression was aberrantly underexpressed in GC cell lines. After the validation of GATA6-AS1 expression in GC, we silenced and overexpressed GATA6-AS1 in GC cells separately to evaluate its loss-of-function and gain-of-function effects on cell viability, proliferation, and migration in GC. Through functional assays, we discovered that overexpressed GATA6-AS1 restrained cell proliferation and migration. In contrast, knockdown of GATA6-AS1 has the promoting effects in the progression of GC. Thus, we confirmed that GATA6-AS1 acts as a tumor suppressor in GC. In normal tissues, this tumor suppressor gene might prevent GC by slowing or stopping cell growth.

Interestingly, GATA6-AS1 was proved to be located in the nucleus and exerted its regulatory function at the transcriptional level in GC [13]. In our study, we discovered that GATA6-AS1 was distributed in both nucleus and cytoplasm through subcellular fractionation and FISH assays. Thus, we speculated that GATA6-AS1 could regulate GC progression through the ceRNA network. In recent years, emerging researches have proven that ceRNA networks play critical roles in the regulation of various cancers [15, 27]. Also, lncRNAs have been confirmed to modulate cancers via acting as a ceRNA of miRNA and regulate mRNA expression at the posttranscriptional level [19].

As a kind of endogenous small noncoding RNA, miRNAs feature 19~26 nucleotides in length [28]. Increasing studies have indicated that lncRNAs combine with miRNAs to regulate cellular progression in cancers. Herein, our study, for the first time, verified and confirmed miR-543 as the target of GATA6-AS1 in GC.

As for miR-543, it has been extensively studied in a number of different human cancers previously. For example, miR-543 was proved to accelerate the proliferation and metastasis of colorectal cancer by targeting KLF4 [29]. Additionally, miR-543 targets RKIP to accelerate cell proliferation and EMT in prostate cancer [30]. Besides, miR-543 can motivate the proliferative and invasive abilities of non-small-cell lung cancer cells [31]. Remarkably, miR-543 has also been validated to promote gastric tumorigenesis [32, 33]. In our research, rescue assays demonstrated that GATA6-AS1 regulates GC cell proliferation and migration via miR-543. Accordingly, this study verified that miR-543 serves as an oncogene in GC.

Moreover, PTEN has been elucidated as the downstream target of miR-543 in multiple cancers, such as colorectal cancer [34, 35]. Herein, our study firstly identified PTEN as a target of miR-543 in GC. PTEN is a dual phosphatase featuring both protein and lipid phosphatase activities and is taken as a tumor suppressor [36]. PTEN exhibits its inhibitory effect on both heritable and sporadic cancers [22]. Besides, the mutation/depletion of PTEN was considered as a negative regulator of the AKT signaling pathway [37]. Also, it was reported that PTEN inhibits PTK6 oncogenic signaling in prostate cancer [38]. In addition, mammary carcinogenesis was confirmed to be associated with PTEN loss of activity rather than loss of expression [39]. Furthermore, some therapeutic strategies targeting PTEN-deficient cancers are under development [40]. In our study, the positive correlation between PTEN and GATA6-AS1 in GC was identified. According to the previous study, AKT signaling pathway is crucial in regulating GC [41, 42]. In our research, western blot indicated that phosphorylated AKT level was reduced by the overexpression of GATA6-AS1 but upregulated by the downregulation of PTEN or addition of SF1670 (PTEN inhibitor). In addition, rescue assays demonstrated that GATA6-AS1 regulates GC progression through the regulation of PTEN/AKT signaling pathway axis.

When it comes to the future research focus in the further studies, the upstream mechanism of GATA6-AS1 in GC will be investigated. For instance, the future investigation will be centered on potential mechanisms underlying the interaction between GATA6-AS1 and its transcription factors since the inactivation of GATA6-AS1 transcription might result in the downregulation of GATA6-AS1 in GC cells. For the downstream mechanism of GATA6-AS1 in GC, other increased miRNAs upon knockdown of GATA6-AS1 as shown in Figure 3(d), such as miR-525-5p, miR-3121-3p, and miR-4424, deserve to be studied to determine whether they are potential targets and how they exert their functions.

Taken together, the current study proved that GATA6-AS1 is lowly expressed in GC tissues and cell lines. More importantly, GATA6-AS1 can repress cell viability, proliferation, and migration in GC by sponging miR-543 and regulating the PTEN/AKT signaling axis. Thus, GATA6-AS1 can act as a potential target for GC treatment, which might provide novel insight into developing effective treatment strategies for patients with GC.

## Data Availability

The data used to support the findings of this study are included within the article.

## Supplementary Materials

Supplementary Materials Figure S1: (A–C) representative image, tumor growth curve, and tumor weight at the end points of xenografted tumors formed by hypodermic injection of stably transfected GC cells into the nude mice. The number of nude mice used in each group is 3. (D) The expression of GATA6-AS1/miR-543/PTEN in xenografted tumor was displayed. (E) The protein expression of PTEN in xenografted tumor was shown. GAPDH was used as an internal reference. One-way ANOVA followed by Dunnett's test. ^∗∗^*P* < 0.01. n.s.: no significance.
