# Supplementary figures and images for: GATA6-AS1 via Sponging miR-543 to Regulate PTEN/AKT Signaling Axis Suppresses Cell Proliferation and Migration in Gastric Cancer
Source: Mediators Inflamm. 2023 May 26;2023:9340499. doi: 10.1155/2023/9340499 (PMC10238141; doi:10.1155/2023/9340499)

A

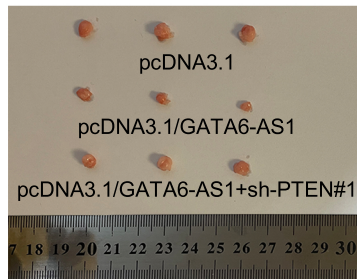

B

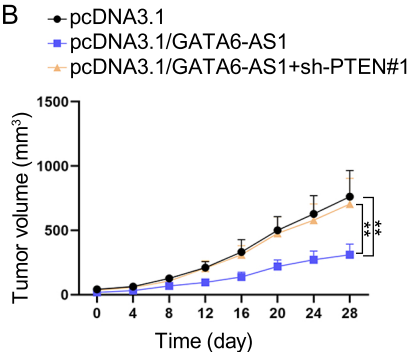

C

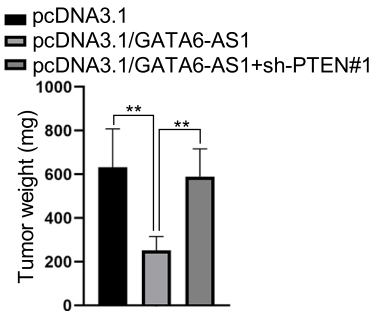

D

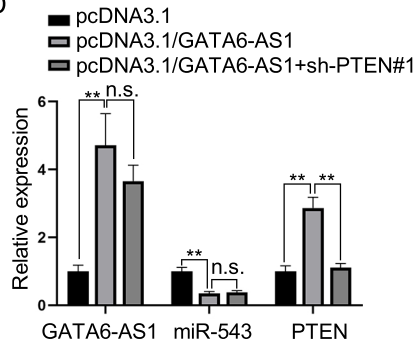

E

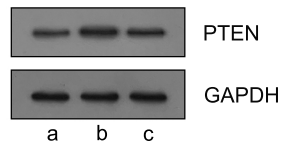

a ■ pcDNA3.1  
b ■ pcDNA3.1/GATA6-AS1  
c ■ pcDNA3.1/GATA6-AS1+sh-PTEN#1

Supplement: Supplementary Materials — Figure S1: (A–C) representative image, tumor growth curve, and tumor weight at the end points of xenografted tumors formed by hypodermic injection of stably transfected GC cells into the nude mice. The number of nude mice used in each group is 3. (D) The expression of GATA6-AS1/miR-543/PTEN in xenografted tumor was displayed. (E) The protein expression of PTEN in xenografted tumor was shown. GAPDH was used as an internal reference. One-way ANOVA followed by Dunnett's test. ∗∗P < 0.01. n.s.: no significance. [file 9340499.f1.pdf]
